# Criteria for site selection in industry-sponsored clinical trials: a survey among decision-makers in biopharmaceutical companies and clinical research organizations

**DOI:** 10.1186/s13063-019-3790-9

**Published:** 2019-12-11

**Authors:** Tilde Dombernowsky, Merete Haedersdal, Ulrik Lassen, Simon Francis Thomsen

**Affiliations:** 1 Department of Dermatology, Copenhagen University Hospital Bispebjerg, Bispebjerg Bakke 23, entrance 9, DK-2400 Copenhagen NV, Denmark; 2 Department of Oncology, Copenhagen University Hospital Rigshospitalet, Blegdamsvej 9, entrance 5073, DK-2100 Copenhagen Ø, Denmark; 3 Department of Biomedical Sciences, University of Copenhagen, Blegdamsvej 3b, DK-2200 Copenhagen N, Denmark

**Keywords:** Trial site selection, Survey, Clinical trials, Pharmaceutical industry, Clinical research organizations

## Abstract

**Background:**

Knowledge of what the pharmaceutical industry emphasizes when assessing trial sites during site selection is sparse. A better understanding of this issue can improve the collaboration on clinical trials and increase knowledge of how to attract and retain industry-sponsored trials. Accordingly, we investigated which site-related qualities multinational biopharmaceutical companies and clinical research organizations (CROs) find most important during site selection.

**Methods:**

An online survey among decision-makers for trial site selection in the Nordic countries employed at multinational biopharmaceutical companies and CROs was conducted. The respondents’ experiences with and perceptions of site selection were addressed to evaluate the relative importance of site-related qualities. We included up to four respondents per company, representing different geographic regions. Descriptive statistics were used to summarize findings.

**Results:**

Of 49 eligible companies, 20 biopharmaceutical companies and 23 CROs participated. In total, 83 responses were analyzed (estimated response rate 78%). A relative importance of site-related qualities was identified: For example, 88% (binomial 95% confidence interval [CI] ±7%) preferred reaching enrollment goals at trial sites in their region 10% quicker rather than cutting the costs at all sites by 20%. Likewise, 42% (CI ±11%) of the respondents preferred that trial sites were best at having the first patients ready for inclusion right after site initiation visit compared to having good data entry, documentation, and reporting practice (25% [CI ±9%]), easily reachable site personnel and backup (23% [CI ±9%]), fast contractual procedure times (6% [CI ±5%]), a key opinion leader associated with the site (3% [CI ±4%]), and updated equipment and facilities (1% [CI ±2%]). In total, 75% [CI ±9%] agreed that their company would be interested in cooperating with an inexperienced trial site if the site had access to a large patient population and 52% [CI ±11%] had experienced that their company selected an inexperienced trial site in favor of an experienced site due to a higher level of interest and commitment.

**Conclusions:**

This study indicates that recruitment-related factors are pivotal to the pharmaceutical industry when assessing trial sites during site selection. Data quality-related factors seem highly valued especially in early phase trials whereas costs and investigator’s publication track record are less important. Experience in conducting clinical trials is not imperative. However, this applies primarily to late phase trials.

## Background

When the pharmaceutical industry assesses potential trial sites during trial site selection, multiple aspects are considered. Factors such as patient population availability, resources at the site, and data collection procedures are evaluated. Likewise, site personnel-related qualities such as interest and commitment, communicative skills, and experience in conducting clinical trials are taken into account. Today, site management is often handled by clinical research organizations (CROs) as many clinical trials are outsourced [1]. Consequently, CROs play a pivotal role during site selection alongside the affiliates of biopharmaceutical companies.

Knowledge of what the pharmaceutical industry emphasizes when selecting European trial sites is sparse; to our knowledge, only two publicly available studies have investigated this [2, 3]. They indicate that recruitment-related factors are pivotal whereas costs are less important. Moreover, they suggest that experience in conducting clinical trials is not imperative.

A better understanding of what the pharmaceutical industry emphasizes when assessing trial sites during site selection can improve the collaboration and performance in clinical trials, ultimately leading to improved medical care. Moreover, a better understanding of this issue can extend knowledge of how trial sites can attract and retain industry-sponsored trials. Accordingly, we conducted a survey among decision-makers for trial site selection in biopharmaceutical companies and CROs to further explore this area.

The aim of this study was to investigate which site-related qualities multinational biopharmaceutical companies and CROs find most important during site selection and while running clinical trials in the Nordic countries. In continuation of the findings by Gehring et al. [3] and findings we made in an interview study conducted in 2016 [2], we particularly focused on recruitment-related factors, costs, and experience in conducting clinical trials. Three main assumptions generated from this previous research were explored:

1. Biopharmaceutical companies and CROs find that recruitment-related factors (i.e. patient population availability, timely patient recruitment, and startup time) are the most important factors during site selection and while running clinical trials;
2. Experience in conducting clinical trials is not imperative to biopharmaceutical companies and CROs when selecting clinical trial sites;
3. The costs of running a clinical trial are secondary to biopharmaceutical companies and CROs if trial sites recruit the patients agreed upon in a timely matter.

## Methods

### Identification of companies and respondents

**Fig. 1:**
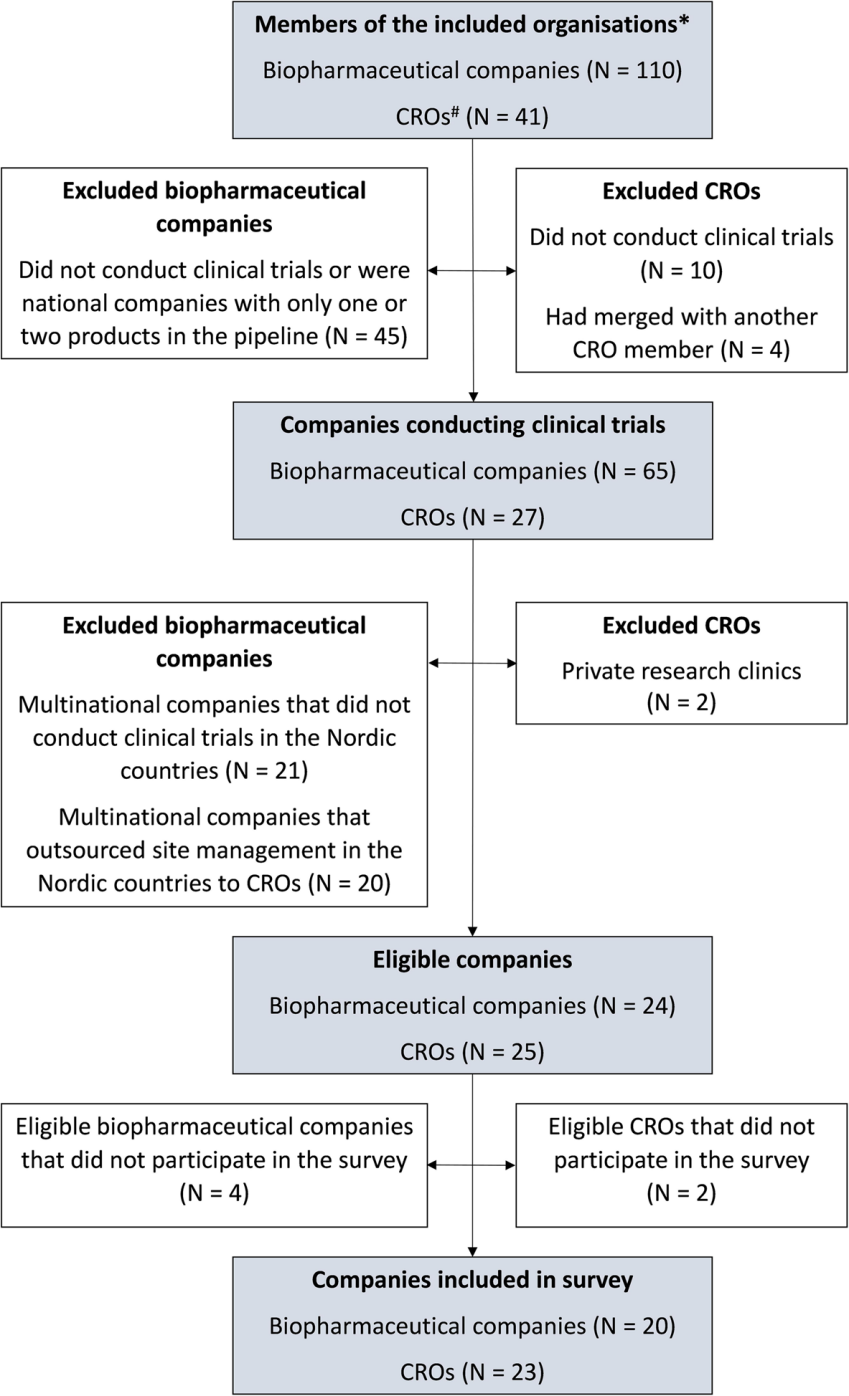
*Flow chart* showing the identification of eligible companies * The Danish Association of the Pharmaceutical Industry, The Swedish Association of the Pharmaceutical Industry, The association for the pharmaceutical industry in Norway, Pharma Industry Finland, The trade association and forum for clinical research organizations active in Sweden, The CRO network of Trial Nation Denmark. # CRO clinical research organization

Our recruitment strategy focused on personal contacts to ensure that relevant companies and respondents were included. First, we identified companies involved in trial site selection in one or more Nordic countries. Thereafter, we identified suitable respondents within each company. Figure 1 illustrates the company selection process.

The following inclusion criteria for the companies were set:

1. Multinational biopharmaceutical company or CRO;
2. Conducted clinical trials in one or more Nordic countries;
3. The affiliate(s) / local office(s) of the company were involved in trial site selection in one or more Nordic countries;
4. Member of one of the following organizations: The Danish Association of the Pharmaceutical Industry; The Swedish Association of the Pharmaceutical Industry; the association for the pharmaceutical industry in Norway; Pharma Industry Finland; the trade association and forum for clinical research organizations active in Sweden; and the CRO network of Trial Nation Denmark.

The following inclusion criteria for the respondents were set:

1. Employed at one of the included companies at a Nordic affiliate / local office;
2. Decision-maker for trial site selection in one or more Nordic countries or involved in the recommendation of trial sites to the sponsor(s).

Using the trial registry ClinicalTrials.gov [4], we estimated that the member companies of the included organizations sponsor or are collaborators in 79% of all industry-sponsored clinical trials conducted in the Nordic countries (Additional file 1). Consequently, we believe that we included the majority of companies involved in trial site selection in the Nordic countries.

Eligible companies and respondents were identified through contact with the Nordic and European affiliate(s) or office(s) by email or phone. A contact person—who in most cases was also a respondent—was sent a link to the online survey and forwarded the link to other eligible participants within the company. We included up to four respondents per company, representing different geographic regions (Denmark, Finland, Norway, and Sweden) as decision-makers for trial site selection employed at the same company may have different perceptions on site selection depending on the region in which they operate. Respondents were recruited continuously during the whole survey response period from 8 May to 8 October 2018. The period was expanded for 1.5 months due to the summer holidays. Because of the recruitment design, the identity of most respondents was known to the authors. However, the respondents were assured that the results would be published without any disclosure of their identity. No remuneration was provided but a summary of the survey results before publication was offered. Additional information on the recruitment process and survey distribution is displayed in the Additional file 1.

### Content of the survey

The survey was a web-based questionnaire addressing the respondents’ perceptions of factors that influence trial site selection in the Nordic countries. Some items aimed at the respondents’ personal opinions, whereas others aimed at the overall opinion of their company. The survey consisted of a background information section followed by three main sections and was completed in 10–15 min using the SurveyXact online platform [5]. The items were presented primarily in Likert scale, single response, and ranking format. In the first section, respondents were asked to indicate their level of agreement with different statements using a five-point scale (strongly agree, agree, undecided, disagree, strongly disagree). In the second section, the respondents’ own experiences with site selection at their company were addressed using primarily single response questions; in the last section, ranking questions were used to evaluate which site-related qualities are the most important in different situations. To avoid missing data, all questions had to be answered before continuing to the next section. To minimize response bias, response categories of the ranking questions were randomly ordered for each respondent individually.

**Fig. 2:**
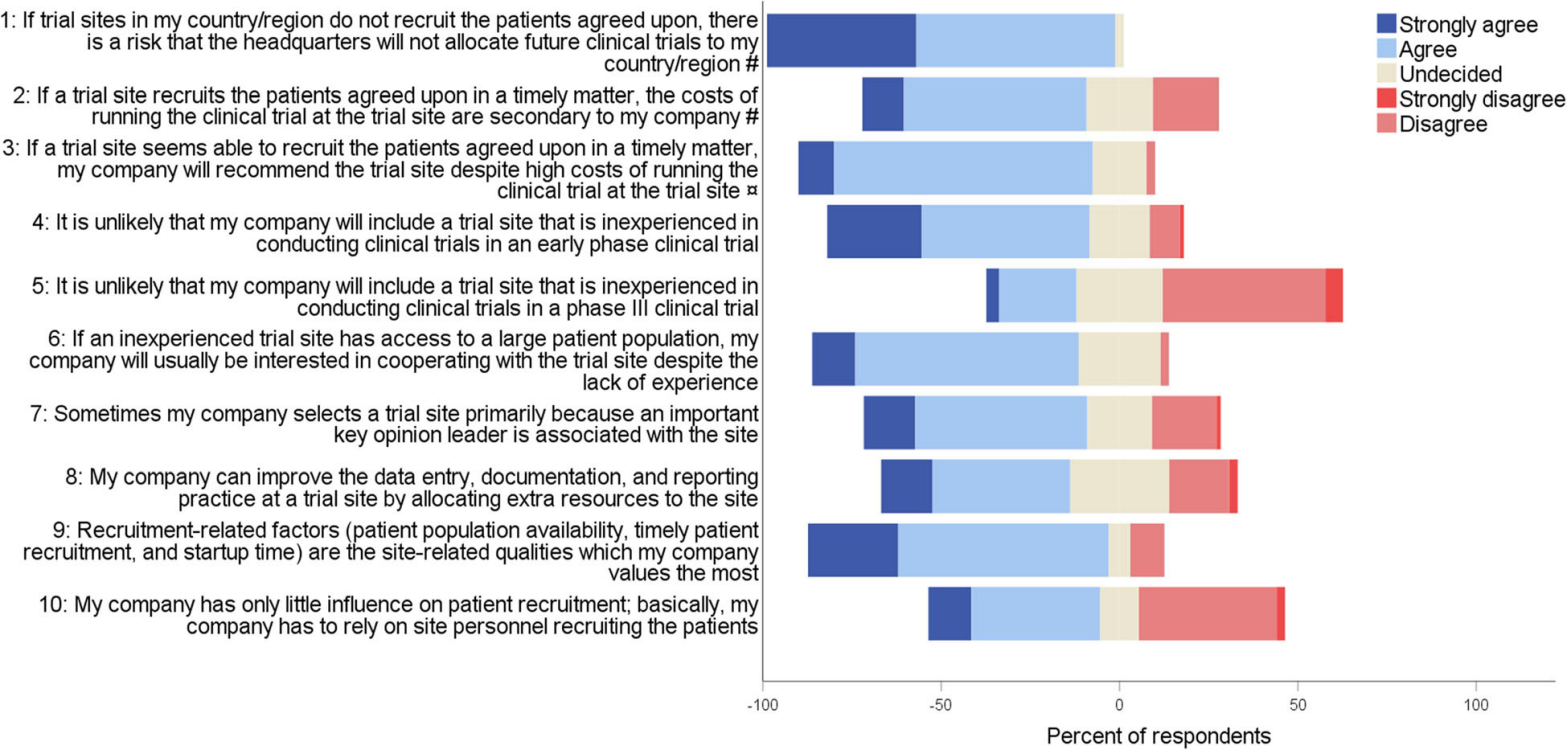
Levels of agreement with statements about trial site selection in the Nordic countries # This question applied to only biopharmaceutical respondents (*n* = 43) ¤ This question applied to only CRO respondents (*n* = 40). CRO clinical research organization

Due to differences in the organizational structure and function of the companies, some items had to be differently formulated depending on the respondent being employed at a biopharmaceutical company or a CRO. Therefore, the two respondent groups received a different questionnaire, although the content was almost identical. For example, during pretesting, CRO respondents stressed that CROs are *recommending* trial sites to the sponsor and not *selecting* trial sites. Therefore, the word *selected* was replaced with *recommended* in relevant items as illustrated in Table 1. We believe that the different wording of the items ensured a homogeneous interpretation of each item across the two respondent groups, still making it possible to evaluate the items as one. However, two items were evaluated separately as the wording differed markedly (Table 1, question 5 and 6; Fig. 2, questions 2 and 3). The full survey for biopharmaceutical and CRO respondents, respectively, are displayed in Additional file 1.

**Table 1:** Experiences with selection and deselection of Nordic trial sites during site selection^a^

Site-related quality	Survey question	Response	
		Yes (%)	No (%)
Experience in conducting clinical trials / Interest and commitment	1. Have you experienced that your company selected/recommended an inexperienced trial site in favor of an experienced trial site during site selection due to a higher level of interest and commitment?	52	48
Interest and commitment	2. Have you experienced that your company selected/recommended a trial site unknown to your company in favor of a well-known trial site due to a higher level of interest and commitment?	57	43
Timely patient recruitment / Key opinion leader	3. Have you experienced that your company selected/recommended a trial site despite an insufficient recruitment in prior trials, because a key opinion leader was associated with the site?	63	37
Timely patient recruitment	4. Have you experienced that your company *deselected*/*did not* recommend a trial site that delivered a timely patient recruitment in prior trials, because your company found it difficult to cooperate with the site in those prior trials?	53	47
Costs at the site	5. Have you experienced that the trial sites selected by the affiliate(s) were not approved by the headquarters because the costs of running the trial at the trial sites were too high?^b^	37	63
Costs at the site	6. Have you experienced that your company *did not* recommend trial sites to a sponsor because the costs of running the trial at the trial sites were too high?^c^	25	75

^a^ Respondents (*n* = 83) were asked about their experiences with site selection at the company they worked for. The words *selected* and *deselected* applied to biopharmaceutical respondents whereas the words *recommended* and *did not recommend* applied to clinical research organization respondents

^b^ This question only applied to biopharmaceutical respondents (*n* = 43)

^c^ This question only applied to clinical research organization respondents (*n* = 40)

Two items in the background section served to ensure that the respondent and the company were indeed decision-makers for trial site selection. If this was not confirmed, the respondent was excluded. Further, the respondent’s company email address was requested to verify that the response came from a relevant person, to determine which company was involved, and to avoid duplicate responses.

### Development and validation of the survey

The development of the survey was based on a previous interview study including employees involved in trial allocation at multinational biopharmaceutical companies [2] and other literature within this field [3, 6–12]. First, we developed an exhaustive list of site-related qualities that the pharmaceutical industry potentially considers during site selection. Subsequently, the items of the survey were constructed, repeatedly reviewing the list, and the three main assumptions that we aimed to investigate. The design and content of the survey were discussed among the authors and iteratively with relevant clinical trial stakeholders and two statisticians. The initial items were scrutinized to mitigate ambiguity and identify concepts that needed to be validated during pretesting, such as *early phase clinical trial* and *data quality*. These concepts were listed and systematically reviewed during pretesting. The pretesting included 19 potential respondents employed at different companies and was carried out at meetings lasting 45–75 min, using a standardized procedure. Additional information on the development and validation of the survey is displayed in Additional file 1.

### Statistical analysis and sample size considerations

We used descriptive statistics to summarize findings. Binomial 95% confidence intervals (CIs) were calculated using the equation for the normal approximation for the binomial confidence interval: p ± z_1-α/2_√(p (1-p)/n). To evaluate potential differences in responses across the two respondent groups, we compared responses using Chi-squared tests and Fisher’s exact tests. Ranking questions were evaluated by comparing differences in the number of first rankings within each response category across the two respondent groups. As the number of respondents in each group was small, we also considered the true values observed. Data were analyzed using SPSS Version 25. A *p* value threshold of ≤ 0.05 was considered statistically significant. There were no missing data as all responses were complete. Given the descriptive design and a finite number of respondents, we did not formally estimate a required sample size.

## Results

Of the 49 eligible companies, 20 biopharmaceutical companies (83%) and 23 CROs (92%) participated in the survey (Fig. 1). The number of decision-makers for trial site selection in the Nordic countries varied between the companies that differed markedly in size and organizational structure. A total of 101 responses were received, of which none were duplicate. Six were partial and all excluded as they were < 20% completed. Further, two were excluded as the respondents reported not to be decision-makers for trial site selection. We received more than one response per Nordic country from four companies. Consequently, 10 responses from these companies were excluded randomly using SPSS. In total, 83 responses were analyzed: 43 from biopharmaceutical companies and 40 from CROs. The average number of respondents per company was 1.9 (standard deviation [SD] 1.1), and the estimated response rate was 78% for both respondent groups (see Additional file 1). The respondents’ type of position and level of experience are displayed in Table 2.

**Table 2:** Characteristics of survey respondents

Characteristics	All respondents (*n* = 83)	Respondents from biopharmaceutical companies (*n* = 43)	Respondents from clinical research organizations (*n* = 40)
Type of company^a^ (frequency counts %)			
Large biopharmaceutical company	41 (50)	41 (95)	–
Small/medium-sized biopharmaceutical company	2 (2)	2 (5)	–
Large CRO	16 (19)	–	16 (40)
Small/medium-sized CRO	24 (29)	–	24 (60)
Position (frequency counts %)			
Clinical operations responsible	48 (58)	35 (81)	13 (33)
Study manager	13 (16)	5 (12)	8 (20)
Monitor	22 (26)	3 (7)	19 (47)
Experience (frequency counts %)			
< 2 years	5 (6)	1 (2)	4 (10)
2–5 years	11 (13)	4 (9)	7 (17)
> 5 years	67 (81)	38 (89)	29 (73)

^a^ Company size was defined in accordance with the definition by the European Commission: Small/medium-sized = employees < 250 and turnover < 50 million € globally (*http://ec.europa.eu/growth/smes/business-friendly-environment/sme-definition_da*)

### Recruitment-related factors (assumption 1)

**Fig. 3:**
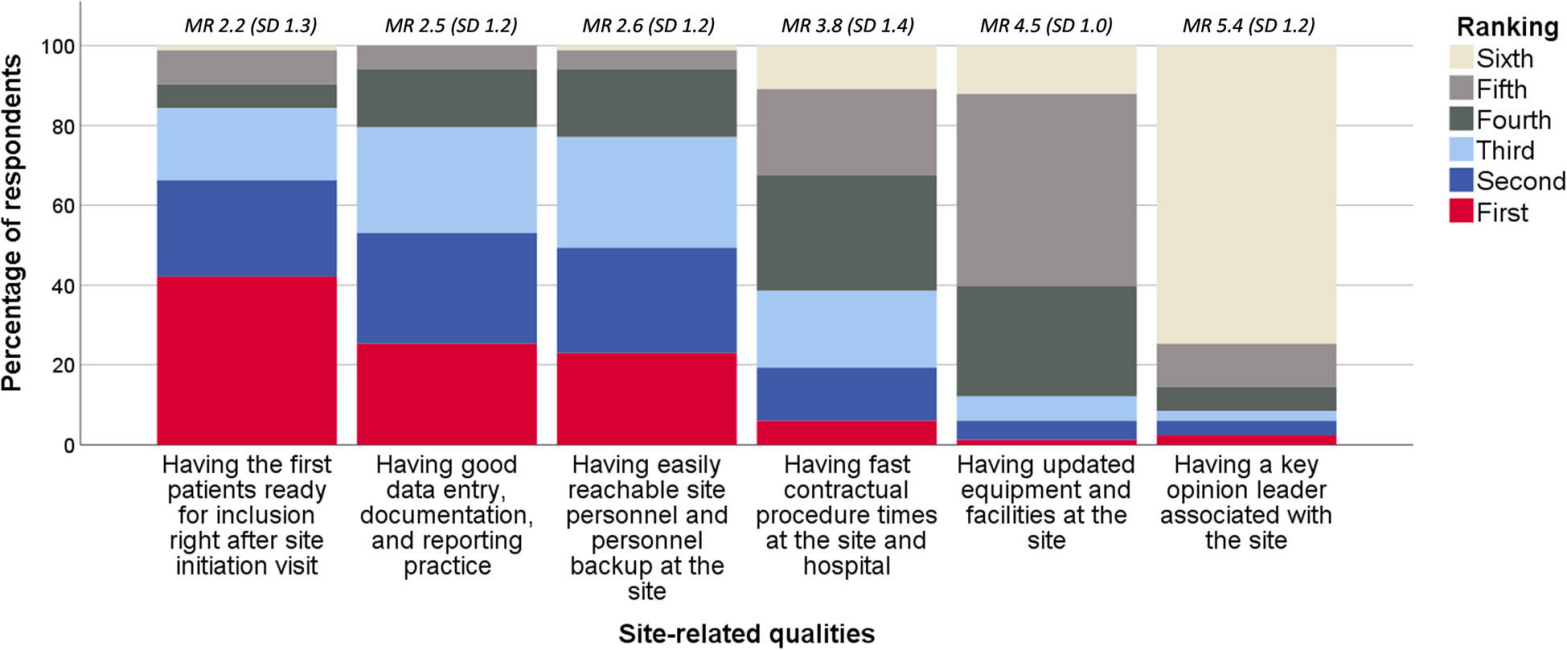
What decision-makers for trial site selection would prefer that Nordic trial sites were best at* * Respondents (*n* = 83) were asked: *If you could choose, what would you prefer that trial sites were best at?* The six response categories were ranked from one to six, one being the most important. MR mean ranking (of the response category), SD standard deviation

In total, 84% (CI ±8%) of the respondents *strongly agreed* or *agreed* that recruitment-related factors are the site-related qualities that their company values the most (Fig. 2, question 9). Likewise, 88% (CI ±7%) preferred reaching enrollment goals at trials sites in their region 10% quicker rather than cutting the costs at all sites by 20% (data not shown). When asked to rank which information about a trial site unknown to their company that the company would find the most valuable, *recruitment and retention track record* was ranked first by 71% (CI ±10%) of the respondents among the six factors tested (Additional file 1: Figure S1). Similarly, when the respondents were asked what they would prefer that trial sites were best at, 42% (CI ±11%) ranked *having the first patients ready for inclusion right after site initiation visit* first (Fig. 3).

**Fig. 4:**
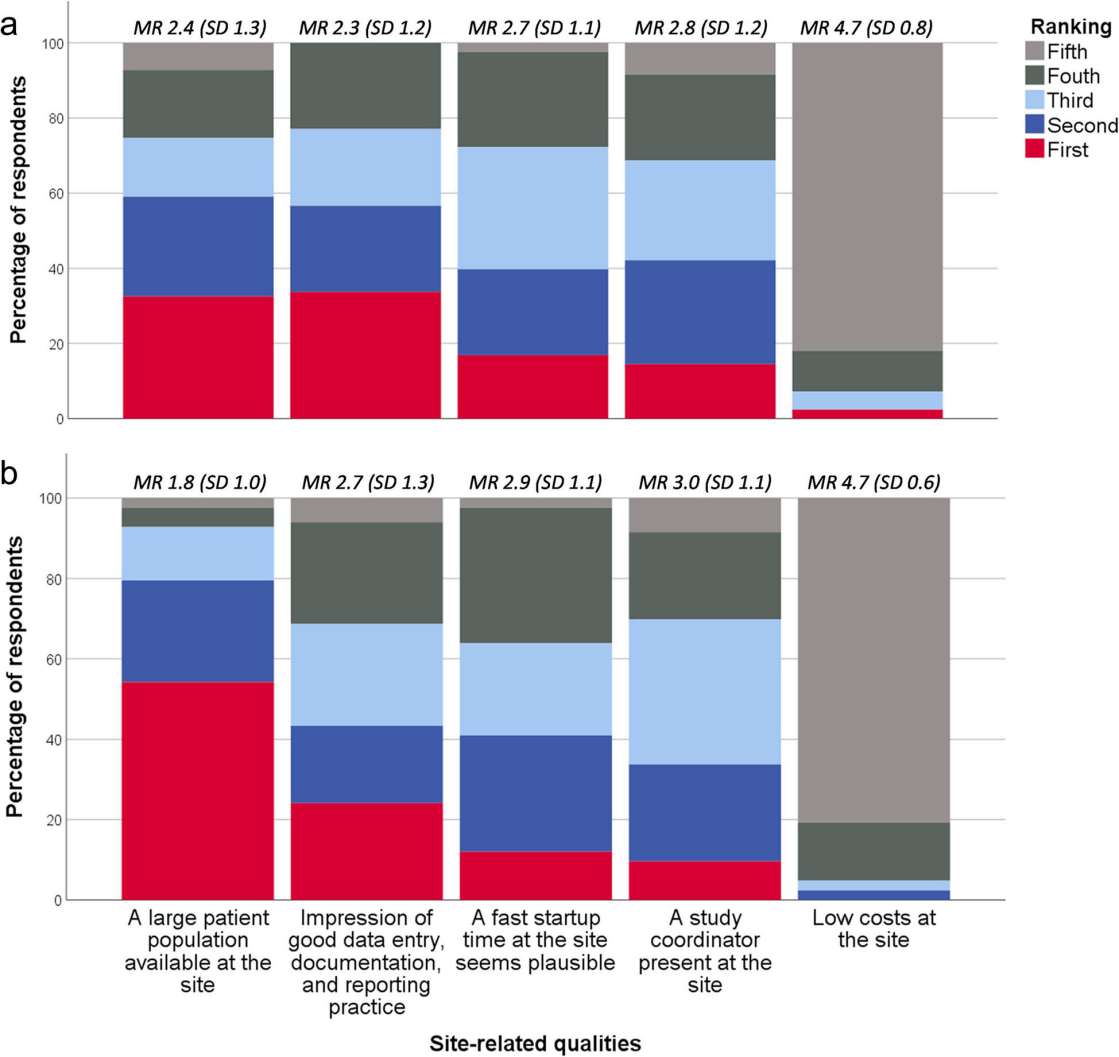
Relative importance of site-related qualities for early phase (**a**) and phase III trials (**b**)* * Respondents (*n* = 83) were asked which of five site-related qualities their company finds the most important during site selection for an early phase clinical trial and phase III clinical trial, respectively. The five response categories were ranked from one to five, one being the most important. MR mean ranking (of the response category), SD standard deviation

**Fig. 5:**
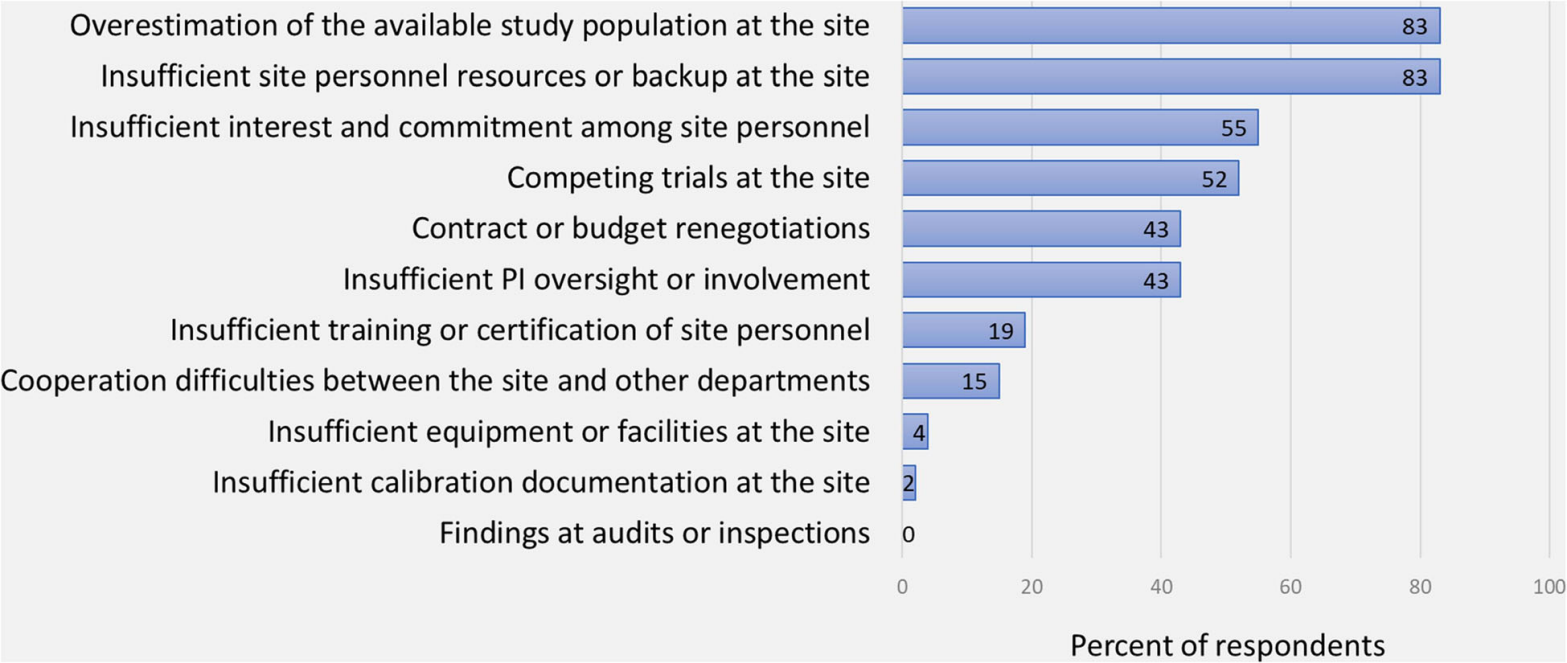
Site-related factors that do most often cause delay in patient recruitment at Nordic trial sites* * Respondents (*n* = 83) were asked to choose among 12 site-related factors the four factors they believe most often cause delay in patient recruitment at the Nordic trial sites that their company cooperates with. Only factors that trial sites influence were included

Figure 4 illustrates the ranking of five site-related qualities according to importance during site selection. For early phase trials, *having a large patient population available at the site* was ranked first by 33% (CI ±10%), whereas it was 54% (CI ±11%) for phase III trials. Two items addressed which of three site-related qualities the clinical operations departments at the affiliates value the most while running an early phase and phase III trial, respectively. *Timely patient recruitment* was ranked the highest in both cases (57% [CI ±11%] and 59% [CI ±11%], respectively) compared to *timely data entry and reporting* (10% [CI ±6%] and 12% [CI ±7%], respectively) and *no critical or major findings at the site during the trial* (33% [CI ±10%] and 29% [CI ±10%], respectively) (Additional file 1: Figure S2). As illustrated by Fig. 5, *overestimation of the available study population* and *insufficient site personnel resources or backup at the site* are the site-related qualities that most often cause delay in patient recruitment at Nordic trial sites according to the respondents.

Two items addressed which factors the headquarters of biopharmaceutical companies find the most important when evaluating the affiliates’ performance and CROs’ performance, respectively, regarding running clinical trials. For both early phase and phase III trials, *timely patient recruitment* was ranked first by most respondents (58% [CI ±11%] and 57% [CI ±11%], respectively) compared to *high data quality* (35% [CI ±10%] and 24% [CI ±9%], respectively), *timely data entry and reporting* (4% [CI ±4%] and 10% [CI ±6%], respectively), and *low costs of running the clinical trial* (3% [CI ±4%] and 9% [CI ±6%], respectively) (Additional file 1: Figure S3).

### Experience in conducting clinical trials (assumption 2)

In total, 75% (CI ±9%) *strongly agreed* or *agreed* that their company would be interested in cooperating with an inexperienced trial site if the trial site had access to a large patient population (Fig. 2, question 6). Further, 52% (CI ±11%) had experienced that their company selected an inexperienced trial site in favor of an experienced site due to a higher level of interest and commitment (Table 1, question 1). In contrast, 74% (CI ±9%) of the respondents *strongly agreed* or *agreed* that it is *un*likely that their company would include an inexperienced trial site for an early phase trial; for phase III trials, it was only 25% (CI ±9%) (Fig. 2, questions 4 and 5).

**Fig. 6:**
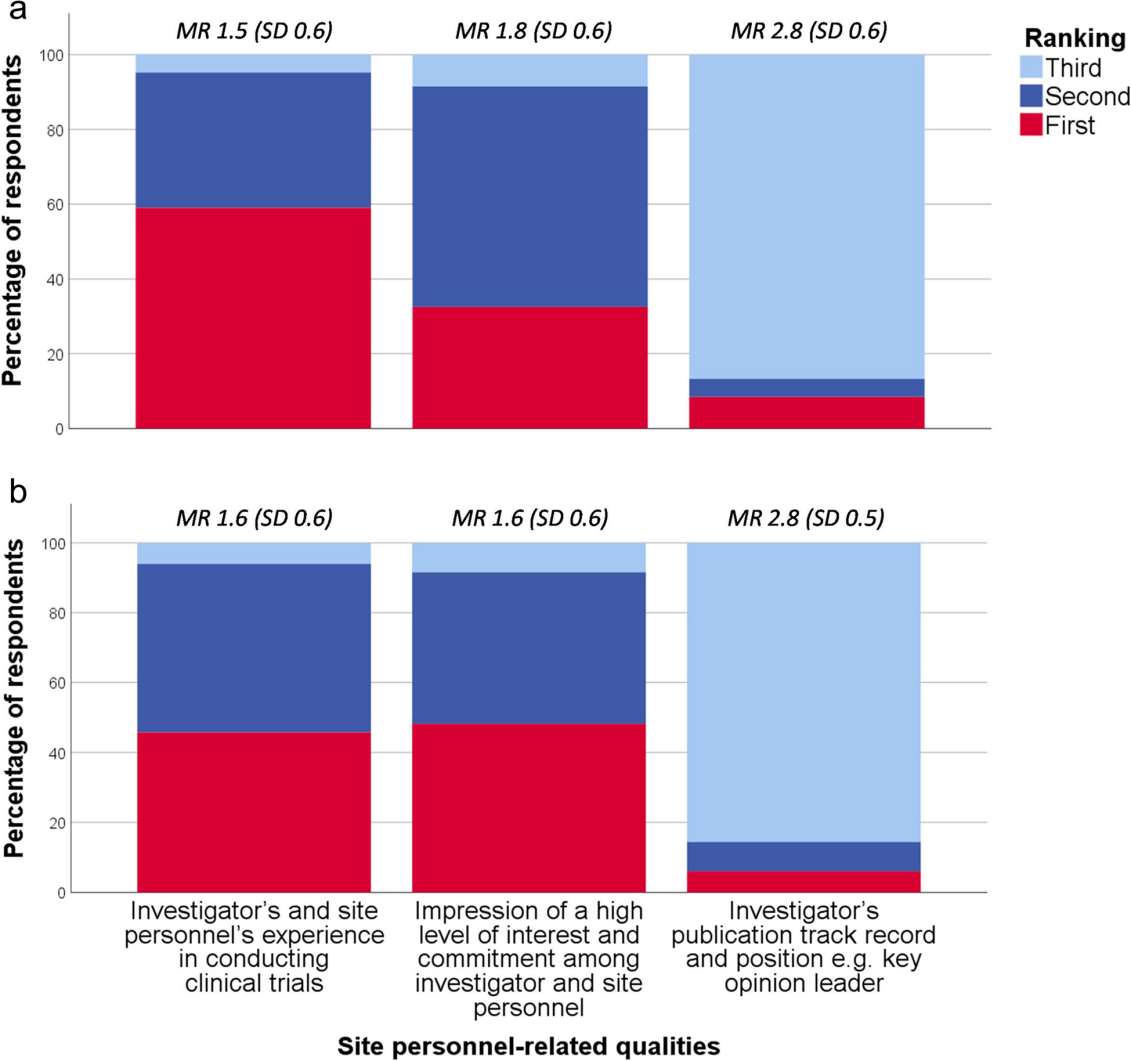
Relative importance of site personnel-related qualities for early phase (**a**) and phase III trials (**b**)* * Respondents (*n* = 83) were asked which of three site personnel-related qualities their company finds the most important during site selection for an early phase clinical trial and phase III clinical trial, respectively. The three response categories were ranked from one to three, one being the most important. MR mean ranking (of the response category), SD standard deviation

Respondents were asked to rank which of three site personnel-related qualities their company finds the most important during site selection: *Experience in conducting clinical trials* was ranked first by 59% (CI ±11%) for early phase trials and 46% (CI ±11%) for phase III trials, whereas *impression of a high level of interest and commitment* was ranked first by 33% (CI ±10%) and 48% (CI ±11%), respectively (Fig. 6). Most respondents believed that if trial site personnel seek out stakeholders at biopharmaceutical companies at conferences displaying a site profile form and track record, the companies would consider including the trial site in future clinical trials: *yes definitely* (24% [CI ±9%]); *yes maybe* (70% [CI ±10%]); and *no* (6% [CI ±5%]).

### Costs (assumption 3)

Most respondents *strongly agreed* or *agreed* that the costs of running a clinical trial at a trial site are secondary if the site recruits the patients agreed upon in a timely matter (Fig. 2, questions 2 and 3). Likewise, when asked which site information is the most valuable to their company, *prices of all trial-related services* was ranked the lowest alongside *data on potential investigators’ publication track record and job position* (Additional file 1: Figure S1). Similarly, *low costs at the site* was ranked lowest among five site-related qualities regarding their importance during site selection (Fig. 4). For both early phase and phase III trials, *low costs of running the clinical trial* was ranked lowest when considering which factors the headquarters find the most important when evaluating the affiliates and CROs (ranked fourth by 70% [CI ±10%] and 63% [CI ±10%], respectively) (Additional file 1: Figure S3).

### Sensitivity analysis

Overall, the response pattern was similar across the two respondent groups. However, more biopharmaceutical than CRO respondents preferred trial sites *having the first patients ready for inclusion right after site initiation visit* (ranked first by 56% [CI ±15%] and 28% [CI ±14%], respectively (*p* = 0.014)) rather than sites *having good data entry, documentation, and reporting practice* (19% [CI ±12%] and 33% [CI ±15%], respectively (*p* = 0.207)). Moreover, notable differences occurred regarding which factors the headquarters of biopharmaceutical companies value the most when evaluating the affiliates’ and CROs’ performance in relation to running clinical trials. *Timely patient recruitment* was ranked first by more biopharmaceutical than CRO respondents for both early phase and phase III clinical trials (65% [CI ±14%] vs 50% [CI ±15%] for early phase trials [*p* = 0.187]; and 74% [CI ±13%] vs 38% [CI ±15%] for phase III trials [*p* = 0.001]). Conversely, *low costs of running the clinical trial* was ranked first by more CRO respondents (8% [CI ±8%] vs 0% of biopharmaceutical respondents for early phase trials [*p* = 0.108]; and 18% [CI ±12%] vs 2% [CI ±5%] for phase III trials [*p* = 0.026]).

## Discussion

In this survey that investigated which site-related qualities the pharmaceutical industry values the most during site selection in the Nordic countries, recruitment-related factors were strongly emphasized, whereas costs and investigator’s publication track record generally had low priority. Data quality-related factors and experience in conducting clinical trials were strongly emphasized in early phase trials, whereas experience was less emphasized in phase III trials.

Recruitment-related factors were highly emphasized throughout the survey for both early phase and phase III clinical trials. This gives weight to the supposition that access to the relevant patient population, a fast startup time, and timely recruitment are among the most important factors when the pharmaceutical industry evaluates trial sites during site selection. Nevertheless, the survey results also indicate that other qualities are sometimes more important. For example, we found that 61% of the respondents had experienced that their company selected a trial site which delivered an insufficient recruitment in prior trials, because a key opinion leader was associated with the site (Table 1, question 3).

According to the respondents, one of the main reasons for insufficient recruitment at Nordic trial sites is *overestimation of the available study population at the site,* when considering factors that trial sites influence. This concurs with findings in our previous interview study in which the participants reported that they often find the investigators’ recruitment projections over-optimistic [2]. Consequently, their company routinely marks down these. This has also been reported by others [13, 14]. Trial sites should take this into consideration and strive to make accurate recruitment projections by carefully considering aspects of the current trial rather than following “gut intuition” or replicating estimations from prior similar trials. That said, the sponsors are also responsible for inaccurate recruitment projections. First, the investigators typically do not have full protocol information when requested to estimate the number of participants the trial site can recruit and the information given by the sponsor changes over time. Second, the response deadline is short, limiting time for a thorough assessment. Third, trial sites are not economically compensated for the time spent which impedes investigators’ motivation to make thorough estimations. As recruitment projections strongly influence study timelines, accurate projections should be of high priority among both trial sites and sponsors to mitigate trial extensions and failure.

Data quality-related factors were generally emphasized less than recruitment-related factors in this survey. One explanation could be that sufficient patient recruitment is crucial to the success of a trial whereas good data quality is not. Another explanation could be that the companies have only little influence on recruitment whereas they can more easily ensure sufficient data quality by allocating extra resources to monitoring and training at the site. However, the results do not confirm this assumption, as responses were ambiguous in this matter (Fig. 2, questions 8 and 10). In our previous interview study, only half of the participants spontaneously mentioned data quality-related factors as important [2]. Moreover, like in this survey, some believed that the headquarters of their company did not value data quality as high as timely patient recruitment. However, when asked, the participants stressed that they find high data quality indispensable. Possibly, these findings reflect that high data quality *is* essential; however, as there are no data without participants, recruitment is emphasized more than data quality during site selection.

Interestingly, the survey results suggest that biopharmaceutical companies and CROs are interested in collaborating with inexperienced trial sites if they have access to the relevant patient population and show interest and commitment. Moreover, interest and commitment is supposedly as important as experience in conducting clinical trials during selection for phase III trials. This concurs with findings by Gering et al. [3] who asked 341 different clinical trial stakeholders to divide 100 points across five investigator-related qualities when selecting trial sites for a phase III/IV trial (*investigator recruitment/retention track record*, *experience in previous trials*, *interest*, *concurrent workload*, and *publication track record*). They found that *interest* was rated as high as *experience in previous trials* (mean 22.4 [SD 13.4] and 22.7 [SD 12.0], respectively). In accordance with our study, *investigator’s publication track record* was least important. The Danish Association of the Pharmaceutical Industry (LIF DK) has also found that commitment is important during site selection. In 2015, LIF DK asked their member companies to describe which site-related qualities they emphasize for early phase trials (personal correspondence with LIF DK). It was stressed that site personnel’s expertise, dedication, and availability are particularly important. Additionally, it was mentioned that the member companies often cooperate with the same preferred trial sites in early phase trials which makes it challenging for inexperienced trial sites to gain cooperation on early phase trials. This is in line with the results of our survey and previous interview study [2] that propose that experience in conducting clinical trials is more important during selection for early phase that late phase trials. This is unsurprising, as early phase trials are usually operationally complex and demand a high level of expertise.

Our results clearly indicate that costs are less important than other factors during site selection, which concurs with previous findings [2, 3, 8]. Nonetheless, this does not necessarily mean that costs are unimportant; costs may play an essential role during country selection, thereby indirectly influencing site selection. Interestingly, our results suggest that costs are of higher influence when the headquarters evaluate the performance of CROs than the performance of their own affiliates. Given the fact that CROs are external partners, this is unsurprising.

We believe that trial sites that already meet the site personnel and facilities requirements necessary to be considered for selection may benefit from emphasizing three aspects in particular during site selection: (1) a thorough and sound assessment of the patient population available at the site; (2) a high level of interest and commitment among site personnel; and (3) a good data entry, documentation, and reporting practice. Further, trial sites that wish to attract industry-sponsored clinical trials will possibly benefit from seeking out stakeholders from the pharmaceutical industry displaying a site profile form and track record. Trial sites should keep in mind that the recruitment performance at one trial site influences the allocation of trials to all sites in the region as the headquarters of biopharmaceutical companies may not allocate future trials to a region delivering an insufficient patient recruitment.

### Strengths and limitations

We believe that this study displays interesting and credible findings. The internal validity of the study is high as the survey was thoroughly constructed and pretested; the respondents were individuals with good reading comprehension who use similar terminology. However, the study has limitations. The number of respondents included in this survey was low; fewer companies than expected were involved in trial site selection in the Nordic countries and several companies had only one primary decision-maker for all Nordic countries. Nevertheless, the respondents were highly representative of the population that we wanted to investigate and the response rate was high. Moreover, the survey included most companies involved in trial site selection in the Nordic countries. To ensure sufficient survey completion, we had to strictly limit the completion time. Consequently, relevant items were omitted which limits the interpretation of the results. Additionally, some site-related qualities were not evaluated. For example, a company’s prior experience with a site is important when selecting trial sites [13, 15]. However, the importance of a good working relationship with the site or site personnel having the right mindset is difficult to evaluate in a quantitative setting. We suspected that all qualities would be rated as highly important if they were simply rated individually. Instead we used ranking questions to assess the relative importance of the site-related qualities. However, this method may lead to more “satisficing” behaviour as rank ordering potential responses is a higher level cognitive task.

## Conclusions

The present study indicates that recruitment-related factors are pivotal to the pharmaceutical industry when assessing trial sites during site selection. Data quality-related factors seem highly valued especially in early phase trials, whereas costs and investigator’s publication track record are generally less important. Experience in conducting clinical trials is not imperative; biopharmaceutical companies and CROs are supposedly interested in cooperating with inexperienced trial sites if they have access to the relevant patient population. However, this applies primarily to late phase trials.

This is one of the first studies investigating which qualities at a trial site the pharmaceutical industry values the most when deciding which trial sites to preferably cooperate with. Hopefully, the findings will contribute to improved collaboration and performance in industry-sponsored clinical trials and help trial sites gain involvement in these trials. In future studies, it would be highly relevant to explore the investigators’ and trial sites’ perspective. For example, little is known about what motivates investigators and trial sites to conduct clinical trials, and what they emphasize when cooperating with the pharmaceutical industry. This area should be further investigated as it is key to understanding how countries and trial sites can attract and retain industry-sponsored clinical trials as well as how to better the cooperation and performance in clinical trials.

## Supplementary information

**Additional file 1: Figure S1.** Information about trial sites that biopharmaceutical companies and CROs would find most valuable if available* * Respondents (*n* = 83) were asked: *Which information about a trial site that your company has not been cooperating with before would your company find the most valuable if available?* The six response categories were ranked from one to six, one being the most valuable. CRO clinical research organizations, MR mean ranking (of the response category), SD standard deviation. **Figure S2.** Relative importance of site-related qualities while running early phase (A) and phase III trials (B)* * Respondents (*n* = 83) were asked which of three site-related qualities the clinical operations departments at the affiliates of their company find the most important while running an early phase and phase III clinical trial, respectively. The three response categories were ranked from one to three, one being the most important. MR mean ranking (of the response category), SD standard deviation. **Figure S3.** The assessment of biopharmaceutical affiliates and CROs in early phase (A) and phase III trials (B)* * The biopharmaceutical-respondents (*n* = 43) were asked which of four factors the headquarters of their company find the most important when evaluating the affiliates’ performance regarding running clinical trials. For CRO respondents (*n* = 40), the question referred to the headquarters evaluation of the CRO. The four response categories were ranked from one to four, one being the most important. CRO clinical research organization, MR mean ranking (of the response category), SD standard deviation

## Data Availability

The full survey for biopharmaceutical and CRO respondents, respectively, are displayed in Additional file 1. The dataset analyzed during the current study are not publicly available to ensure anonymity of the survey participants but are available from the corresponding author on reasonable request.

## Abbreviations

CROs: Clinical research organizations
LIF DK: The Danish Association of the Pharmaceutical Industry
SD: Standard deviation
