# Supplementary material for: Criteria for site selection in industry-sponsored clinical trials: a survey among decision-makers in biopharmaceutical companies and clinical research organizations
Source: Trials. 2019 Dec 11;20:708. doi: 10.1186/s13063-019-3790-9 (PMC6907246; doi:10.1186/s13063-019-3790-9)
Supplement: Supplementary file 1 — Additional file 1: Figure S1. Information about trial sites that biopharmaceutical companies and CROs would find most valuable if available* * Respondents (n = 83) were asked: Which information about a trial site that your company has not been cooperating with before would your company find the most valuable if available? The six response categories were ranked from one to six, one being the most valuable. CRO clinical research organizations, MR mean ranking (of the response category), SD standard deviation. Figure S2. Relative importance of site-related qualities while running early phase (A) and phase III trials (B)* * Respondents (n = 83) were asked which of three site-related qualities the clinical operations departments at the affiliates of their company find the most important while running an early phase and phase III clinical trial, respectively. The three response categories were ranked from one to three, one being the most important. MR mean ranking (of the response category), SD standard deviation. Figure S3. The assessment of biopharmaceutical affiliates and CROs in early phase (A) and phase III trials (B)* * The biopharmaceutical-respondents (n = 43) were asked which of four factors the headquarters of their company find the most important when evaluating the affiliates’ performance regarding running clinical trials. For CRO respondents (n = 40), the question referred to the headquarters evaluation of the CRO. The four response categories were ranked from one to four, one being the most important. CRO clinical research organization, MR mean ranking (of the response category), SD standard deviation [file 13063_2019_3790_MOESM1_ESM.docx]

**Appendix to ‘Criteria for site selection in industry-sponsored clinical trials:
A survey among decision-makers in biopharmaceutical companies and
clinical research organizations’**

1. [**Additional information on identification of companies and respondents, and survey distribution** 1](#_Toc534571316)

2. [**Additional information on development and validation of the survey**](#_Toc534571318) 2

[3. **Estimation of survey response rates**](#_Toc534571317) 4

4. [**Assessment of sponsorship for industry-sponsored clinical trials in the Nordic countries**](#_Toc534571319) 4

5. [**Additional figures**](#_Toc534571319) 7

6. [**Full text of survey questionnaire for biopharmaceutical-respondents** 10](#_Toc534571321)

7. [**Full text of survey questionnaire for CRO-respondents**](#_Toc534571322) 20

**1. Additional information on identification of companies and respondents,**

**and survey distribution**

To identify eligible companies, we firstly reviewed the websites of all member companies of the included organizations (i.e. The Danish Association of the Pharmaceutical Industry; The Swedish Association of the Pharmaceutical Industry; the association for the pharmaceutical industry in Norway; Pharma Industry Finland; the trade association and forum for clinical research organizations active in Sweden; and the CRO-network of Trial Nation Denmark). Some companies were excluded as they did not conduct clinical trials e.g. companies engaged in aquaculture and consultancies offering merely regulatory associated services. Others were excluded as they were national companies with none or only few products in the pipeline (typically small biotechnology companies) (Figure 1 of the article). Subsequently, we contacted the Nordic or European affiliate(s) or local office(s) of the remaining companies to further investigate eligibility. Of the companies that did conduct clinical trials, some conducted clinical trials only outside the Nordic countries, and others outsourced site management in the Nordic region to CROs who were decision-makers for trials site selection in cooperation with the global headquarters. These companies were excluded as well.

In each of the participating companies, we identified a contact person who in most cases was also a respondent. The contact person forwarded the survey link to other eligible participants within the company. The contact person was requested to estimate the number of decision-makers for trial site selection within the Nordic affiliate(s) or office(s) at the company, and the number of colleagues he or she forwarded the survey to. Thereby, we could estimate a response rate for each company.

**2. Additional information on development and validation of the survey**

In this survey, we focused on site selection for pre-marketing trials only. Early on, we decided to distinguish between early and late stage trials because the site-related qualities valued the most likely vary considerably across these. It was discussed with the test-respondents how they perceived different terms and which they found suitable. Most of the test-respondents preferred the terms *early phase trial* and *phase III trial*. The term *late phase trial* was considered inadequate as it was perceived as referring to both pre- and post-marketing trials.

We found that the best way to assess the relative importance of different site-related qualities was to use several types of questions (primarily Likert scale-, single response, and ranking questions). During previous interviews that we conducted among employees involved in trial allocation at multinational biopharmaceutical companies [1], it was stressed that all site-related qualities are important. Therefore, we suspected that all qualities would be rated as highly important, if they were simply rated individually. Instead, we used forced ranking questions to assess which site-related qualities were the most important in different situations. All response categories were carefully evaluated to ensure that they were representative, and no essential categories were omitted. The number of response categories were restricted to ensure response validity. Certain site-related qualities were considered indisputable as trial sites must meet specific site personnel- and facilities requirements to be even considered for selection. For example, *certification of site personnel; equipment at the site;* and no *competing trials* were considered given by the test-respondents. Consequently, these factors were omitted. Some response categories were changed during pretesting; for example, *updated facilities and equipment at the site* was removed from the item displayed in Figure 4 of the article as the test-respondents considered this quality to be given. Instead, *a study coordinator present at the site* was added as several test-respondents emphasized this as an important quality.

The first draft of the survey was pretested by 7 potential respondents at meetings lasting between 45 and 75 minutes. Thereafter, the items were revised accordingly. The second draft was pretested by 12 potential respondents, also at meetings lasting between 45 and 75 minutes. Subsequently, the final survey was developed. The pretesting was carried out using a standardized procedure. First, the test-respondent filled in the questionnaire online without commenting or asking questions to the first author (TD) who conducted all meetings. Thereafter, the respondent gave overall comments on the design and content of the survey. Subsequently, concept words and phrases were reviewed to clarify if the respondent perceived the concepts in the same way as we did. For example, the respondent was asked: “how do you understand *data quality*?” or “what is an *early phase trial* to you?” Finally, all items were systematically discussed to clarify if these were clear, non-ambiguous, and included relevant and comprehensive response categories. Of the 19 persons who pretested the survey, 11 participated in the final survey. Our previous interview study [1] included 11 participants. Of these, seven participated in the final survey.

**3. Estimation of survey response rates**

The number of decision-makers for trial site selection in the Nordic countries varied between the companies that differed markedly in size and organisational structure. Some companies had only one decision-maker for all Nordic countries whereas others had numerous decision-makers. We included up to four respondents per company representing different geographic regions (Denmark, Finland, Norway, and Sweden). For each company, we assessed the number of potential responses from 1 to 4 based on information from the contact person about the site selection process and number of decision-makers within the company. For example, if a company had one primary decision-maker for trial site selection in Denmark and Norway based at the Danish affiliate, and one primary decision-maker for Sweden and Finland based at the Swedish affiliate, the potential number of responses was set to two. If a company had multiple decision-makers for trial site selection at affiliates in all Nordic countries, the potential number of responses was set to four. If we received responses from the head of clinical operations in 3 of the 4 affiliates, the response rate was set to 75%. The overall response rate of the survey was 77.9% for the biopharmaceutical companies and 77.5% for the CROs.

**4. Assessment of sponsorship for industry-sponsored clinical trials**

**in the Nordic countries**

Using the trial registry ClinicalTrials.gov, we estimated that the member companies of the included organizations sponsor or are collaborators in 79% of all industry-sponsored clinical trials conducted in the Nordic countries. Data were extracted on the 26^th^ June 2018 from the Aggregate Analysis of ClinicalTrials.gov database (AACT), which is a publicly available database that contains all information about every study registered in ClinicalTrials.gov [2]. We identified all industry-sponsored interventional trials with research facilities in Denmark, Finland, Iceland, Norway, or Sweden having a start date between 1^st^ January 2008 and 26^th^ June 2018 (n = 2802). For each trial, we extracted data on the lead sponsor and collaborator sponsor(s). A lead sponsor was registered for all trials. For 659 of the 2802 trials, one or more collaborator sponsors were registered. We organized the trials by name of the lead sponsor and manually reviewed the trials to identify those who had a lead sponsor or collaborator sponsor that were members of the organizations included (i.e. The Danish Association of the Pharmaceutical Industry; The Swedish Association of the Pharmaceutical Industry; the association for the pharmaceutical industry in Norway; Pharma Industry Finland; the trade association and forum for clinical research organizations active in Sweden; and the CRO-network of Trial Nation Denmark). The member companies of the organizations included in the study were lead sponsors or collaborator sponsors in 2202 of the 2802 trials (79%). Consequently, we believe that these organizations include the majority of companies involved in trial site selection in the Nordic countries.

**References**

1. Dombernowsky T, Haedersdal M, Lassen U, Thomsen SF. Clinical trial allocation in multinational pharmaceutical companies - a qualitative study on influential factors. Pharmacology research & perspectives. 2017;5(3):e00317.

2. The Clinical Trials Transformation Initiative (CTTI). Improving Public Access to Aggregate Content of ClinicalTrials.gov. 2017. <https://aact.ctti-clinicaltrials.org/>. Accessed March 2018.**5. Additional figures**

**Figure S1: Information about trial sites that biopharmaceutical companies and CROs would find most valuable if available***


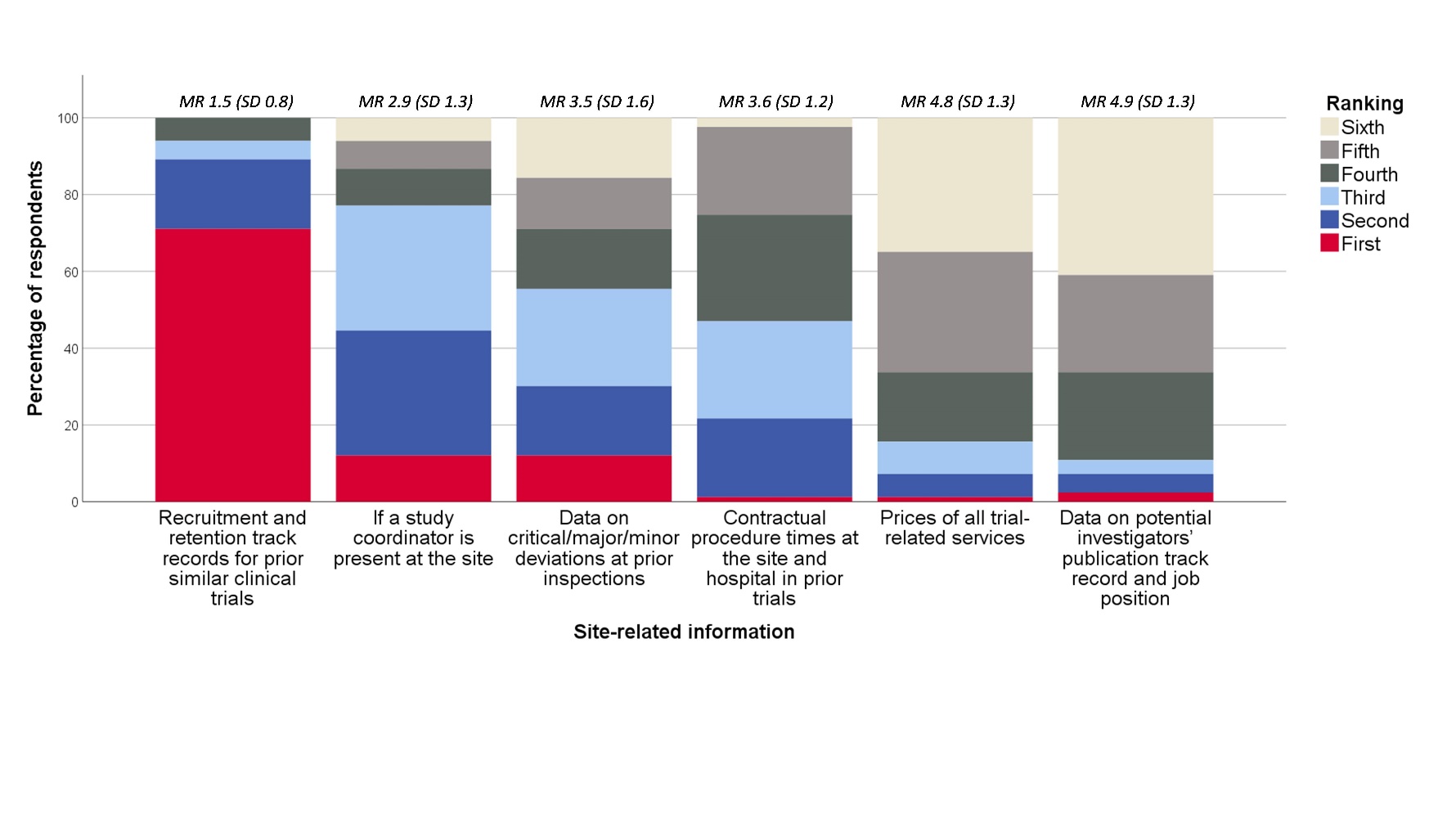


* Respondents (n = 83) were asked: *Which information about a trial site that* *your company has not been cooperating with before would your company find the most valuable if available?* The six response categories were ranked from one to six, one being the most valuable. CROs = clinical research organizations. MR = mean ranking (of the response category). SD = standard deviation.

**Figure S2: Relative importance of site-related qualities
while running early phase (A) and phase III trials (B)***


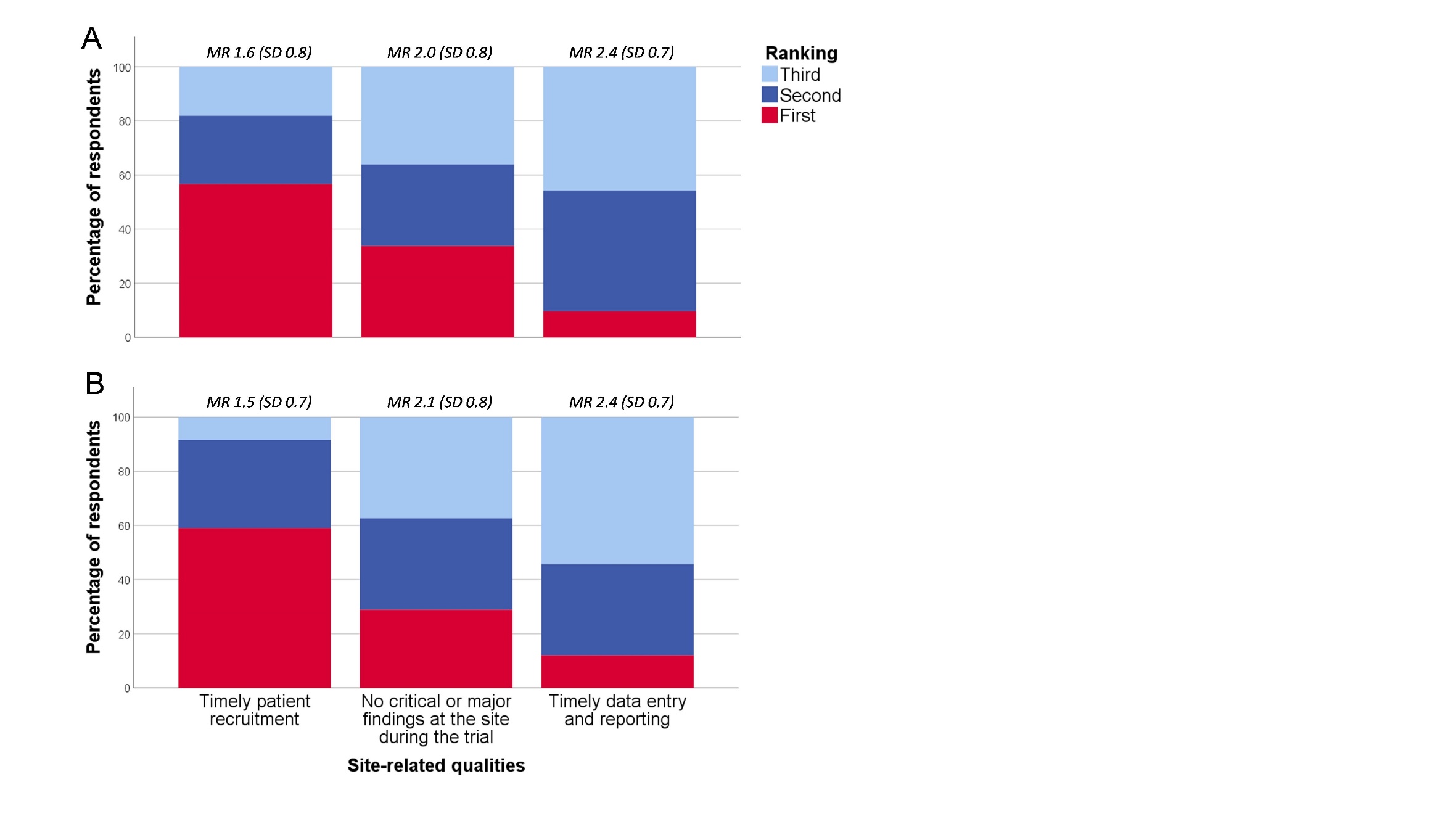


* Respondents (n = 83) were asked which of three site-related qualities the clinical operations departments at the affiliates of their company find the most important while running an early phase and phase III clinical trial, respectively. The three response categories were ranked from one to three, one being the most important. MR = mean ranking (of the response category). SD = standard deviation.

**Figure S3: The assessment of biopharmaceutical affiliates
and CROs in early phase (A) and phase III trials (B)***


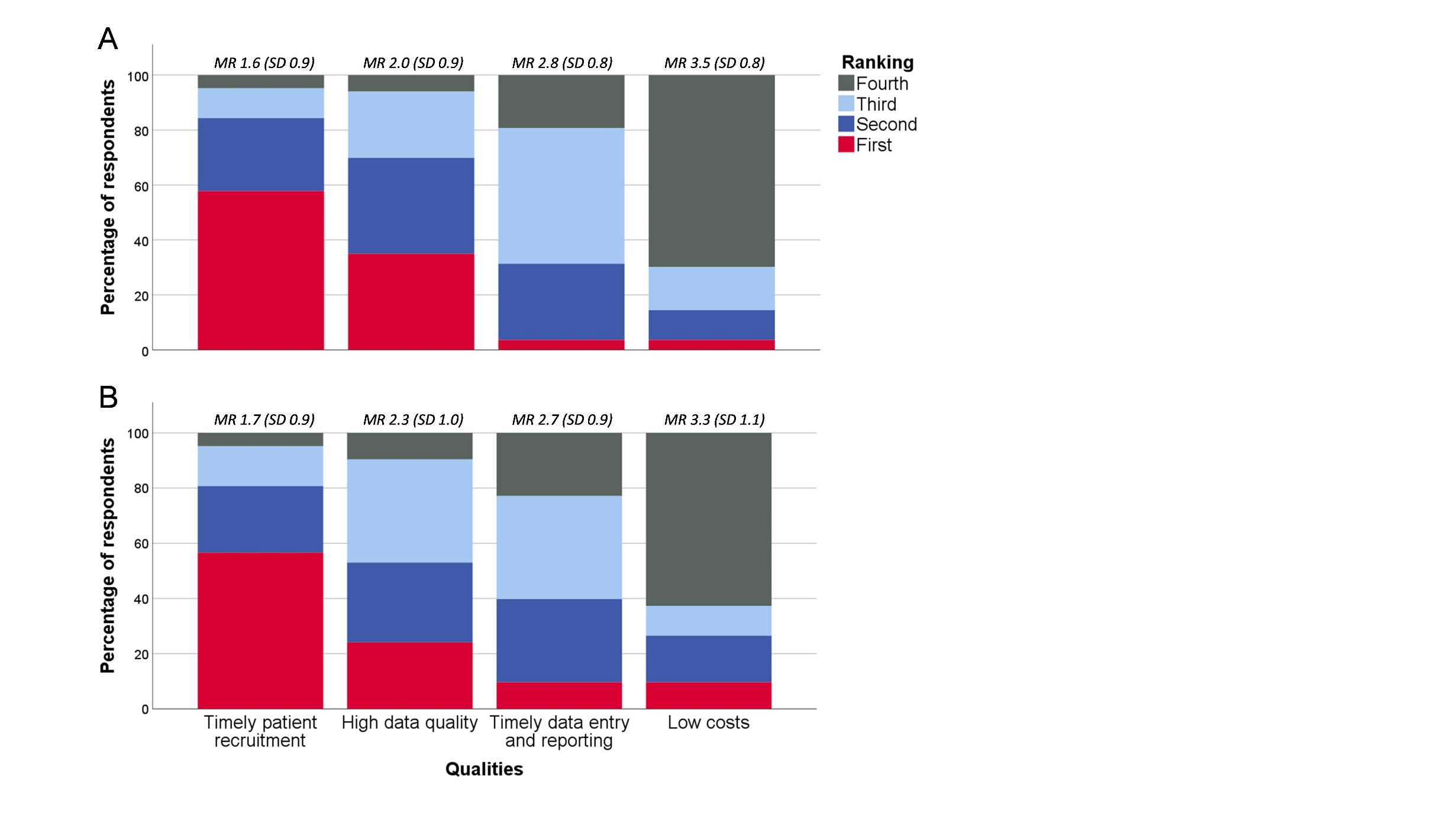


* The biopharmaceutical-respondents (n = 43) were asked which of four factors the headquarters of their company find the most important when evaluating the affiliates’ performance regarding running clinical trials. For CRO-respondents (n = 40), the question referred to the headquarters evaluation of the CRO. The four response categories were ranked from one to four, one being the most important. CROs = clinical research organizations. MR = mean ranking (of the response category). SD = standard deviation.

**Full text of survey questionnaire for biopharmaceutical-respondents:**

**The survey of clinical trial site selection in multinational pharmaceutical companies and clinical research organisations**

**Introduction**

**The aim of this survey** is to investigate which site-related qualities multinational pharmaceutical companies and clinical research organisations (CROs) find most important during site selection and while running clinical trials of medicines at clinical trial sites in the Nordic countries. Hopefully, this will give trial sites a better understanding of on what basis trial sites are selected, thereby improving the collaboration on clinical trials.

The survey is conducted as a part of a Danish PhD project, examining the cooperation between the pharmaceutical industry and clinical trial sites with regard to running clinical trials of medicines.

**The completion time of the questionnaire is 10 to 15 minutes**. The questions concern general perceptions and opinions. Therefore, no detailed or confidential information concerning your company is uncovered. The results will be published without any disclosure of respondents.

**We would appreciate your reply by Monday 8th October 2018.**

You will have the opportunity to receive a summary of the survey results before publication if interested.

**Thank you for your time!**

**Tilde Dombernowsky**

MD, PhD student

Copenhagen University Hospital Bispebjerg,

Department of dermatology, Denmark

Email: dombernowsky@gmail.com

Phone: +45 61661058

**Simon Francis Thomsen**

Professor, MD

Copenhagen University Hospital Bispebjerg,

Department of dermatology, Denmark

**Merete Hædersdal**

Professor, MD

Copenhagen University Hospital Bispebjerg,

Department of dermatology, Denmark

**Ulrik Lassen**

Professor, MD

Copenhagen University Hospital Rigshospitalet

Department of oncology, Denmark

**Background information**

Please verify your email address

***This is solely to confirm that data originate from a relevant clinical trial stakeholder. We will not use your email address for any other purpose.***


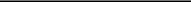


Please indicate which type of company most closely resembles yours (globally).

Small/medium-sized pharmaceutical or biotechnology company*


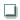


Large pharmaceutical or biotechnology company


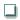


Small/medium-sized clinical research organisation (CRO)*


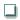


Large clinical research organisation (CRO)


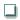


*Employees < 250 and turnover < 50 million EUR

Please indicate the position that most closely resembles yours.

Monitor


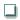


Study manager


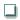


Clinical operations responsible


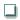


Comments if any:


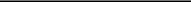


Are you a decision-maker for clinical trial site selection in your country/region (either independently or in cooperation with colleagues)?

Yes


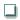


No


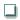


Please indicate for which Nordic country(ies) you are involved in trial site selection.

Denmark


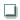


Finland


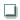


Iceland


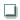


Norway


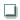


Sweden


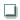


Please indicate your level of experience within the field of clinical trial site selection.

< 2 years of experience


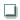


2-5 years of experience


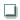


> 5 years of experience


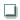


Please indicate to what extent decision-making for clinical trial site selection is outsourced at your company.

Trial site selection is solely my company’s decision


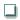


Trial site selection is both my company’s and external partner(s)’ decision Trial site selection is solely external partner(s)’ decision


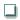

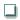


Comments if any:


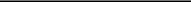


**Please indicate your level of agreement**

**with the following nine statements**

If trial sites in my country/region do not recruit the patients agreed upon, there is a risk that the headquarters will not allocate future clinical trials to my country/region.

Strongly agree


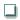


Agree


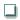


Undecided


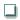


Disagree


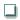


Strongly disagree


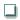


If a trial site recruits the patients agreed upon in a timely matter, the costs of running the clinical trial at the trial site are secondary to my company.

Strongly agree


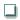


Agree


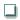


Undecided


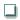


Disagree


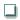


Strongly disagree


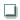


It is unlikely that my company will include a trial site that is inexperienced in conducting clinical trials in **an early phase** clinical trial.

Strongly agree


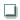


Agree


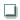


Undecided


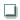


Disagree


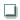


Strongly disagree


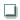


It is unlikely that my company will include a trial site that is inexperienced in conducting clinical trials in **a phase III** clinical trial.

Strongly agree


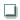


Agree


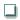


Undecided


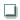


Disagree


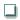


Strongly disagree


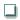


If an inexperienced trial site has access to a large patient population, my company will usually be interested in cooperating with the trial site despite the lack of experience.

Strongly agree


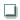


Agree


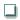


Undecided


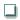


Disagree


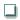


Strongly disagree


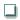


Sometimes my company selects a trial site primarily because an important key opinion leader is associated with the site.

Strongly agree


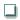


Agree


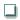


Undecided


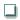


Disagree


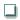


Strongly disagree


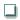


My company can improve the data entry, documentation, and reporting practice at a trial site by allocating extra resources to the site.

Strongly agree


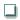


Agree


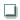


Undecided


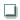


Disagree


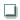


Strongly disagree


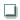


Recruitment related factors (patient population availability, timely patient recruitment, and startup time) are the site-related qualities which my company values the most.

Strongly agree


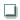


Agree


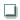


Undecided


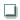


Disagree


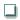


Strongly disagree


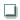


My company has only little influence on patient recruitment; basically, my company has to rely on site personnel recruiting the patients.

Strongly agree


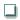


Agree


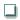


Undecided


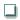


Disagree


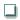


Strongly disagree


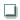


**For the following eight questions, please choose the response category you find the most appropriate**

Have you experienced that your company selected an inexperienced trial site in favor of an experienced trial site during site selection due to a higher level of interest and commitment?

Yes


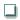


No


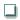


Have you experienced that your company selected a trial site unknown to the company in favor of a well-known trial site due to a higher level of interest and commitment?

Yes


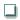


No


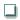


Have you experienced that your company selected a trial site despite an insufficient recruitment in prior trials, because a key opinion leader was associated with the site?

Yes


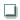


No


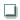


Have you experienced that your company **deselected** a trial site that delivered a timely patient recruitment in prior trials, because your company found it difficult to cooperate with the site in those prior trials?

Yes


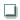


No


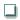


Have you experienced that the trial sites selected by the affiliate(s) were not approved by the headquarters because the costs of running the trial at the trial sites were too high?

Yes


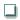


No


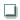


Would you prefer reaching enrollment goals at trial sites in your region 10% quicker or cutting the costs of running the trial at all sites by 20%?

Reaching enrollment goals 10% quicker


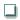


Cutting the costs by 20%


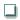


Which of the following factors do most often cause delay in patient recruitment at the Nordic trial sites that your company cooperates with?

**Please choose the four most influential factors (only factors that trial sites influence are included).**

Cooperation difficulties between the site and other departments such as laboratories


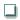


Insufficient PI oversight or involvement


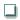


Insufficient site personnel resources or backup at the site


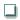


Insufficient equipment or facilities at the site


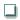


Insufficient training or certification of site personnel


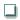


Findings at audits or inspections


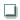


Overestimation of the available study population at the site


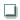


Insufficient calibration documentation at the site


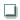


Competing trials at the site


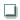


Insufficient interest and commitment among site personnel


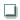


Contract or budget renegotiations


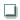


If trial site personnel seek out stakeholders at pharmaceutical companies at conferences displaying a site profile form and track record, do you believe the companies will consider including the trial site in future clinical trials?

Yes, definitely


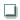


Yes, maybe


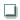


No


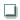


Please elaborate on your answer (optional)

____________

**For the following ten questions, please rank the importance of the response categories with 1 being the most important**

**Drag and drop the categories using the mouse**

**We acknowledge that the determination as to which site-related qualities are most important depends on various factors such as the specific protocol, the therapeutic area, and availability of trial sites. For each question, please think of which qualities your company generally finds most important.**

During site selection for **an early phase** clinical trial, which of the following site-related qualities does your company find the most important?

1. A study coordinator present at the site
2. A fast startup time at the site seems plausible
3. A large patient population available at the site
4. Impression of good data entry, documentation, and reporting practice
5. Low costs at the site

During site selection for **an early phase** clinical trial, which of the following site personnel-related qualities does your company find the most important?

1. Investigator’s and site personnel’s experience in conducting clinical trials
2. Impression of a high level of interest and commitment among investigator and site personnel
3. Investigator’s publication track record and position e.g. key opinion leader

During site selection for **a phase III** clinical trial, which of the following site-related qualities does your company find the most important?

1. Impression of good data entry, documentation, and reporting practice
2. A fast startup time at the site seems plausible
3. A large patient population available at the site
4. A study coordinator present at the site
5. Low costs at the site

During site selection for **a phase III** clinical trial, which of the following site personnel-related qualities does your company find the most important?

1. Impression of a high level of interest and commitment among investigator and site personnel
2. Investigator’s and site personnel’s experience in conducting clinical trials
3. Investigator’s publication track record and position e.g. key opinion leader

Which information about a trial site that your company has not been cooperating with before would your company find the most valuable if available?

1. Contractual procedure times at the site and hospital in prior trials
2. If a study coordinator is present at the site
3. Recruitment and retention track records for prior similar clinical trials
4. Data on potential investigators’ publication track record and job position
5. Data on critical/major/minor deviations at prior inspections
6. Prices of all trial-related services

While running **an early phase** clinical trial, which of the following site-related qualities do the clinical operations departments at the affiliates of your company find the most important?

1. Timely data entry and reporting
2. No critical or major findings at the site during the trial
3. Timely patient recruitment

While running **a phase III** clinical trial, which of the following site-related qualities do the clinical operations departments at the affiliates of your company find the most important?

1. Timely patient recruitment
2. Timely data entry and reporting
3. No critical or major findings at the site during the trial

When the headquarters of your company evaluates **an** **early phase** clinical trial with regard to the affiliates’ performance, which of the following factors do you believe it finds the most important?

1. High data quality
2. Timely patient recruitment
3. Low costs of running the clinical trial
4. Timely data entry and reporting

When the headquarters of your company evaluates **a phase**

- 1. clinical trial with regard to the affiliates’ performance, which of the following factors do you believe it finds the most important?

1. Timely patient recruitment
2. High data quality
3. Low costs of running the clinical trial
4. Timely data entry and reporting

If you could choose, what would you prefer that trial sites were best at?

1. Having good data entry, documentation, and reporting practice
2. Having fast contractual procedure times at the site and hospital
3. Having updated equipment and facilities at the site
4. Having easily reachable site personnel and personnel backup at the site
5. Having a key opinion leader associated with the site
6. Having the first patients ready for inclusion right after site initiation visit

In your opinion, what should Nordic trial sites primarily focus on if they want to ensure that pharmaceutical companies keep allocating clinical trials to their trial site?

____________

Please provide any comments you may have here.

____________

If you wish to receive a summary of the survey results before publication, please indicate the e-mail-address to which they should be sent.

____________

**Thank you for your time!**

**Full text of survey questionnaire for CRO-respondents:**

**The survey of clinical trial site selection in multinational pharmaceutical companies and clinical research organisations**

**Introduction**

**The aim of this survey** is to investigate which site-related qualities multinational pharmaceutical companies and clinical research organisations (CROs) find most important during site selection and while running clinical trials of medicines at clinical trial sites in the Nordic countries. Hopefully, this will give trial sites a better understanding of on what basis trial sites are selected, thereby improving the collaboration on clinical trials.

The survey is conducted as a part of a Danish PhD project, examining the cooperation between the pharmaceutical industry and clinical trial sites with regard to running clinical trials of medicines.

**The completion time of the questionnaire is 10 to 15 minutes**. The questions concern general perceptions and opinions. Therefore, no detailed or confidential information concerning your company is uncovered. The results will be published without any disclosure of respondents.

**We would appreciate your reply by Monday 8th October 2018.**

You will have the opportunity to receive a summary of the survey results before publication if interested.

**Thank you for your time!**

**Tilde Dombernowsky**

MD, PhD student

Copenhagen University Hospital Bispebjerg,

Department of dermatology, Denmark

Email: dombernowsky@gmail.com

Phone: +45 61661058

**Simon Francis Thomsen**

Professor, MD

Copenhagen University Hospital Bispebjerg,

Department of dermatology, Denmark

**Merete Hædersdal**

Professor, MD

Copenhagen University Hospital Bispebjerg,

Department of dermatology, Denmark

**Ulrik Lassen**

Professor, MD

Copenhagen University Hospital Rigshospitalet

Department of oncology, Denmark

**Background information**

Please verify your email address

***This is solely to confirm that data originate from a relevant clinical trial stakeholder. We will not use your email address for any other purpose.***


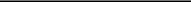


Please indicate which type of company most closely resembles yours (globally).

Small/medium-sized pharmaceutical or biotechnology company*


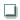


Large pharmaceutical or biotechnology company


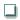


Small/medium-sized clinical research organisation (CRO)*

Large clinical research organisation (CRO)

*Employees < 250 and turnover < 50 million EUR

Please indicate the position that most closely resembles yours.

Monitor

Study manager

Clinical operations responsible

Comments if any:

Is your company usually involved in the assessment and/or selection of trial sites for the clinical trials that your company is involved in?

Yes

No, assessment and selection of trial sites is solely made by the sponsor

Are you involved in the assessment and/or selection of trial sites for the clinical trials that your company is involved in?

Yes

No

Please indicate for which Nordic country(ies) you are involved in trial site selection.

Denmark

Finland

Iceland

Norway

Sweden

Please indicate your level of experience within the field of clinical trial site selection.

< 2 years of experience

2-5 years of experience

> 5 years of experience

**Throughout the questionnaire, please think of the recommendation of trial sites as the process where your company assesses potential trial sites during feasibility and pre-study visits and thereafter recommends trial sites to the sponsor for final selection.**

**Please indicate your level of agreement**

**with the following eight statements**

If a trial site seems able to recruit the patients agreed upon in a timely matter, my company will recommend the trial site despite high costs of running the clinical trial at the trial site.

Strongly agree

Agree

Undecided

Disagree

Strongly disagree

It is unlikely that my company will recommend a trial site that is inexperienced in conducting clinical trials for **an early** **phase** clinical trial.

Strongly agree

Agree

Undecided

Disagree

Strongly disagree

It is unlikely that my company will recommend a trial site that is inexperienced in conducting clinical trials for **a** **phase III** clinical trial.

Strongly agree

Agree

Undecided

Disagree

Strongly disagree

If an inexperienced trial site has access to a large patient population, my company will usually be interested in cooperating with the trial site despite the lack of experience.

Strongly agree

Agree

Undecided

Disagree

Strongly disagree

Sometimes my company recommends a trial site primarily because an important key opinion leader is associated with the site.

Strongly agree

Agree

Undecided

Disagree

Strongly disagree

My company can improve the data entry, documentation, and reporting practice at a trial site by allocating extra resources to the site.

Strongly agree

Agree

Undecided

Disagree

Strongly disagree

Recruitment related factors (patient population availability, timely patient recruitment, and startup time) are the site-related qualities which my company values the most.

Strongly agree

Agree

Undecided

Disagree

Strongly disagree

My company has only little influence on patient recruitment; basically, my company has to rely on site

personnel recruiting the patients.

Strongly agree

Agree

Undecided

Disagree

Strongly disagree

**For the following eight questions, please choose the response category you find the most appropriate**

Have you experienced that your company recommended an inexperienced trial site in favor of an experienced trial site due to a higher level of interest and commitment?

Yes

No

Have you experienced that your company recommended a trial site unknown to your company in favor of a well-known trial site due to a higher level of interest and commitment?

Yes

No

Have you experienced that your company recommended a trial site despite an insufficient recruitment in prior trials, because a key opinion leader was associated with the site?

Yes

No

Have you experienced that your company **did not** recommend a trial site that delivered a timely patient recruitment in prior trials, because your company found it difficult to cooperate with the site in those prior trials?

Yes

No

Have you experienced that your company **did not** recommend trial sites to a sponsor because the costs of running the trial at the trial sites were too high?

Yes

No

Would you prefer reaching enrollment goals at trial sites in your region 10% quicker or cutting the costs of running the trial at all sites by 20%?

Reaching enrollment goals 10% quicker

Cutting the costs by 20%

Which of the following factors do most often cause delay in patient recruitment at the Nordic trial sites that your company cooperates with?

**Please choose the four most influential factors (only factors that trial sites influence are included).**

Insufficient site personnel resources or backup at the site

Cooperation difficulties between the site and other departments such as laboratories

Contract or budget renegotiations

Insufficient equipment or facilities at the site

Insufficient calibration documentation at the site

Findings at audits or inspections

Overestimation of the available study population at the site

Competing trials at the site

Insufficient PI oversight or involvement

Insufficient training or certification of site personnel Insufficient interest and commitment among site personnel

If trial site personnel seek out stakeholders at pharmaceutical companies at conferences displaying a site profile form and track record, do you believe the companies will consider including the trial site in future clinical trials?

Yes, definitely

Yes, maybe

No

Please elaborate on your answer (optional)

_____________

**For the following ten questions, please rank the importance of the response categories with 1 being the most important**

**Drag and drop the categories using the mouse**

**We acknowledge that the determination as to which site-related qualities are most important depends on various factors such as the specific protocol, the therapeutic area, and availability of trial sites. For each question, please think of which qualities your company generally finds most important.**

During site selection for **an early phase** clinical trial, which of the following site-related qualities does your company find the most important?

1. A study coordinator present at the site
2. A large patient population available at the site
3. Impression of good data entry, documentation, and reporting practice
4. Low costs at the site
5. A fast startup time at the site seems plausible

During site selection for **an early phase** clinical trial, which of the following site personnel-related qualities does your company find the most important?

1. Investigator’s and site personnel’s experience in conducting clinical trials
2. Impression of a high level of interest and commitment among investigator and site personnel
3. Investigator’s publication track record and position e.g. key opinion leader

During site selection for **a phase III** clinical trial, which of the following site-related qualities does your company find the most important?

1. A fast startup time at the site seems plausible
2. A study coordinator present at the site
3. A large patient population available at the site
4. Low costs at the site
5. Impression of good data entry, documentation, and reporting practice

During site selection for **a phase III** clinical trial, which of the following site personnel-related qualities does your company find the most important?

1. Investigator’s publication track record and position e.g. key opinion leader
2. Investigator’s and site personnel’s experience in conducting clinical trials
3. Impression of a high level of interest and commitment among investigator and site personnel

Which information about a trial site that your company has not been cooperating with before would your company find the most valuable if available?

1. If a study coordinator is present at the site
2. Contractual procedure times at the site and hospital in prior trials
3. Data on critical/major/minor deviations at prior inspections
4. Data on potential investigators’ publication track record and job position
5. Prices of all trial-related services
6. Recruitment and retention track records for prior similar clinical trials

While running **an early phase** clinical trial, which of the following site-related qualities do the clinical operations departments at the affiliates of your company find the most important?

1. Timely data entry and reporting
2. Timely patient recruitment
3. No critical or major findings at the site during the trial

While running **a phase III** clinical trial, which of the following site-related qualities do the clinical operations departments at the affiliates of your company find the most important?

1. Timely patient recruitment
2. No critical or major findings at the site during the trial
3. Timely data entry and reporting

When sponsors evaluate **early phase** clinical trials with regard to your company’s performance, which of the following factors do you believe they find the most important?

1. Low costs of running the clinical trial
2. Timely data entry and reporting
3. High data quality
4. Timely patient recruitment

When sponsors evaluate **phase III** clinical trials with regard to your company’s performance, which of the following factors do you believe they find the most important?

1. Low costs of running the clinical trial
2. Timely patient recruitment
3. Timely data entry and reporting
4. High data quality

If you could choose, what would you prefer that trial sites were best at?

1. Having the first patients ready for inclusion right after site initiation visit
2. Having fast contractual procedure times at the site and hospital
3. Having a key opinion leader associated with the site
4. Having easily reachable site personnel and personnel backup at the site
5. Having good data entry, documentation, and reporting practice
6. Having updated equipment and facilities at the site

In your opinion, what should Nordic trial sites primarily focus on if they want to ensure that pharmaceutical companies keep allocating clinical trials to their trial site?

____________

Please provide any comments you may have here.

___________

If you wish to receive a summary of the survey results before publication, please indicate the e-mail-address to which they should be sent.

___________

**Thank you for your time!**
